# Potential Roles of Tumor Cell- and Stroma Cell-Derived Small Extracellular Vesicles in Promoting a Pro-Angiogenic Tumor Microenvironment

**DOI:** 10.3390/cancers12123599

**Published:** 2020-12-02

**Authors:** Nils Ludwig, Dominique S. Rubenich, Łukasz Zaręba, Jacek Siewiera, Josquin Pieper, Elizandra Braganhol, Torsten E. Reichert, Mirosław J. Szczepański

**Affiliations:** 1 Department of Oral and Maxillofacial Surgery, University Hospital Regensburg, 93053 Regensburg, Germany; nils.ludwig@ukr.de (N.L.); torsten.reichert@ukr.de (T.E.R.); 2 Programa de Pós-Graduação em Biociências, Universidade Federal de Ciências da Saúde de Porto Alegre (UFCSPA), Porto Alegre 90050-170, Brazil; dominiquesr@ufcspa.edu.br (D.S.R.); ebraganhol@ufcspa.edu.br (E.B.); 3 Department of Biochemistry, Medical University of Warsaw, 02-091 Warsaw, Poland; s068791@student.wum.edu.pl; 4 Department of Hyperbaric Medicine, Military Institute of Medicine, 04-141 Warsaw, Poland; jsiewiera@wim.mil.pl; 5 Department of Oral and Maxillofacial Surgery, University Hospital Bochum, 44892 Bochum, Germany; josquin.pieper@kk-bochum.de

**Keywords:** tumor-derived exosomes, small extracellular vesicles, angiogenesis, cancer, tumor microenvironment

## Abstract

**Simple Summary:**

In this review, we focus on the distinct functions of tumor-cell-derived small extracellular vesicles in promotion of angiogenesis and describe their potential as a therapeutic target for anti-angiogenic therapies. Also, we focus on extracellular vesicles derived from non-cancer cells and their potential role in stimulating a pro-angiogenic tumor microenvironment. The article describes the biogenesis of small extracellular vesicles and refers to their proteomic cargo components that play a role in promoting angiogenesis. Moreover, we explain how small extracellular vesicles derived from tumors and non-cancer cells can interact with recipient cells and alter their functions. We particularly focus on phenotypical and functional changes in endothelial cells, macrophages, and neutrophils that result in proangiogenic signaling.

**Abstract:**

Extracellular vesicles (EVs) are produced and released by all cells and are present in all body fluids. They exist in a variety of sizes, however, small extracellular vesicles (sEVs), the EV subset with a size range from 30 to 150 nm, are of current interest. They are characterized by a distinct biogenesis and complex cargo composition, which reflects the cytosolic contents and cell-surface molecules of the parent cells. This cargo consists of proteins, nucleic acids, and lipids and is competent in inducing signaling cascades in recipient cells after surface interactions or in initiating the generation of a functional protein by delivering nucleic acids. Based on these characteristics, sEVs are now considered as important mediators of intercellular communication. One hallmark of sEVs is the promotion of angiogenesis. It was shown that sEVs interact with endothelial cells (ECs) and promote an angiogenic phenotype, ultimately leading to increased vascularization of solid tumors and disease progression. It was also shown that sEVs reprogram cells in the tumor microenvironment (TME) and act in a functionally cooperative fashion to promote angiogenesis by a paracrine mechanism involving the differential expression and secretion of angiogenic factors from other cell types. In this review, we will focus on the distinct functions of tumor-cell-derived sEVs (TEX) in promotion of angiogenesis and describe their potential as a therapeutic target for anti-angiogenic therapies. Also, we will focus on non-cancer stroma-cell-derived small extracellular vesicles and their potential role in stimulating a pro-angiogenic TME.

## 1. Introduction

Small extracellular vesicles (sEVs) are a fraction of the extracellular vesicles produced by all cell types, including tumor cells [1,2]. They are described as small membranous vesicles with diameters ranging from 30 to 150 nm, that carry selected proteins, lipids, nucleic acids, and glycoconjugates. Growing evidence suggests that sEVs are present in all body fluids such as plasma, cerebrospinal fluid, urine, and saliva [1,3]. They are produced during the inward invagination of the endosome which results in the formation of small vesicles, called intraluminal bodies (ILVs), encapsulated in a larger multivesicular body (MVB). After the maturation and transport of the MVB to the peripheral areas of the cell, it fuses with the cell membrane and releases sEVs into the extracellular space [4]. sEVs were considered to function as cellular bins, but research in recent years has brought evidence that they play a crucial role in intercellular communication under physiological and pathological conditions [5]. In particular, sEVs produced by tumor cells, thus tumor-derived sEVs (TEX), gained a lot of interest due to their role in tumor growth, metastasis, immune escape, and angiogenesis [6,7]. Growing evidence suggests that cancer cells release larger quantities of sEVs compared to non-cancer cells and, therefore, TEX are enriched in the tumor microenvironment (TME) and plasma of cancer patients [8]. This enhanced release of TEX was shown to be associated with cellular stress, such as hypoxia, acidic pH, and many other triggers present in the TME [9,10,11]. Due to their large amount in biofluids, sEVs were proposed as useful biomarkers for non-invasive cancer monitoring in the context of a liquid biopsy [2]. Among the above-mentioned functions, the stimulation of tumor angiogenesis appears to be one hallmark of TEX and it is crucial for tumor persistence and progression [12]. It was shown that TEX carry a plethora of angiogenic factors and molecules, which can contribute to the formation of new blood vessels [6]. In this review, we focus on the role of TEX in promoting a pro-angiogenic TME. Furthermore, we discuss stroma non-cancer cell-derived sEVs and their contribution to the promotion of angiogenesis with special regard to endothelial cells, macrophages, and neutrophils.

## 2. Biogenesis of sEVs

The biogenesis of sEVs begins with the formation of an early endosome (EE) by inward invagination of the plasma membrane [5]. Subsequently, the EE maturates into a late endosome (LE), which begins to produce intraluminal vesicles (ILVs) by using an endosomal sorting complex required for transport (ESCRT) [13,14]. ILVs originate from inward budding of the endosomal membrane, which encloses fragments of cytosolic content and transmembrane and peripheral proteins into smaller vesicles located in the endosome. A newly-formatted endosome enriched in luminar ILVs is considered a multivesicular body (MVB) [15]. Finally, mature MVBs can fuse with the lysosome membrane and become involved in the degradation pathway or fuse with the cell membrane, which results in sEV release into the extracellular fluid (ECF) [15].

The formation of ILVs and their sorting process in MVBs is defined by a highly complicated mechanism, which is precisely regulated by the ESCRT complex. This complex consists of four proteins—ESCRT-0, ESCRT-I, ESCRT-II, and ESCRT-III [16,17]. ILV formation begins with sorting and sequestrating of ubiquitinated proteins by ESCRT-0 in specific sections of the MVB membrane [18]. Afterwards, ESCRT-I and ESCRT-II connect with ESCRT-0 and assemble a complex with high avidity for the ubiquitinated proteins. Subsequently, ESCRT-III joins to the rest of the complex and induces membrane deformation, thereby commencing the inward invagination and ILV formation [19]. Finally, newly-originated ILVs are released into the MVB lumen. Now, the ubiquitinated ILV cargo is destined for lysosomal degradation unless deubiquitylating enzymes (DUBs) change this fate. TSG101 which is an ESCRT-machinery component when ubiquitinated with ISG15 (ISGylated) can trigger the MVB aggregation with lysosomes and its protein degradation [20]. Interestingly, in some conditions deubiquitylation of TSG101 may induce lysosomal trafficking [16]. Moreover, ATPase VPS4 dissociates the ESCRT protein complex from the membrane and enables its recycling [21,22,23].

**Figure 1:**
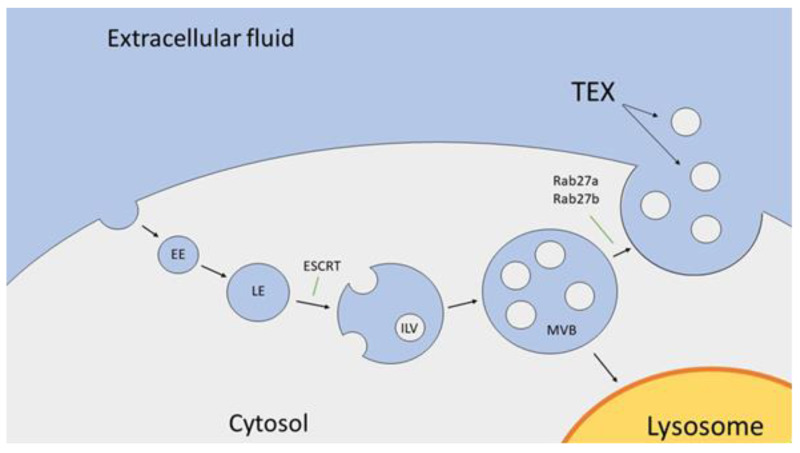
Biogenesis and secretion of small extracellular vesicles. The EE is created by inward invagination of the cell membrane. Afterwards, the EEs maturate into LEs. The ESCRT molecular machinery is involved in producing ILVs leading to the formation of MVBs, which contain many ILVs in its lumen. MVBs undergo one of two main pathways, since they (1) fuse with a lysosome or (2) degrade or fuse with the cell membrane and release small extracellular vesicles (sEVs). Abbreviations: EE, early endosome; LE, late endosome; ESCRT, endosomal sorting complex required for transport; ILV, intraluminal vesicle; MVB, multivesicular body; and TEX, tumor-derived sEVs.

Mature MVBs can directly fuse with the lysosome and deliver its content for a degradation or target the cell membrane and release ILVs, from what are now called sEVs, into the ECF. Rab27a and Rab27b are important regulators of MVB transport to the peripheral areas of the cell and promote its docking with the cell membrane, ultimately releasing sEVs into the ECF [24]. sEVs are secreted by virtually all types of cells, however, cancer cells show outstanding activity in sEV production when compared to non-cancer cells [5]. The underlying mechanisms of sEV production in different cell types, with special regards to potential discriminations between cancer and non-cancer cells, are still to be determined. Uncovering the precise molecular interactions and pathways of sEV production is part of ongoing research. The biogenesis of sEVs is illustrated in Figure 1.

## 3. Proteomic Cargo of TEX

TEX play a key role in mediating intercellular communication by transporting proteins, lipids, nucleic acids, and many other molecules involved in signaling processes from cancer cells to recipient cells [25,26,27]. TEX are enriched in a variety of factors, such as proteases, enzymes, growth factors, and cytokines, which are transported in the lumen of TEX and are delivered to different recipient cells in the TME, facilitating tumor growth and expansion [28]. Moreover, TEX carry cargo components on their surface such as receptors/ligands, adherent molecules, or tetraspanins (e.g., CD63, CD9, and CD81) [29]. The molecular cargo composition of TEX reflects the contents of the donor cell [30], however, recent evidence suggests that some cargo components of TEX are a result of a special sorting mechanism rather than an exact reflection of donor cell composition [31]. Furthermore, the sphingosine 1-phosphate receptor was suggested to play a role in distributing molecules to ILVs [32].

**Figure 2:**
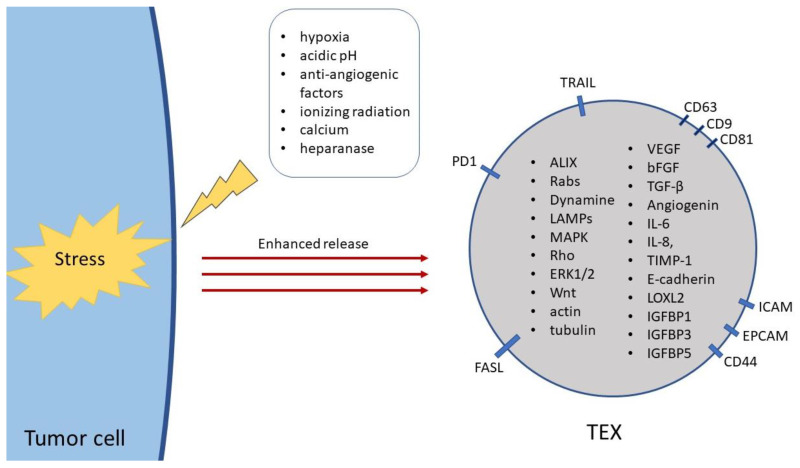
Release of TEX and their molecular cargo composition. Stress conditions such as hypoxia, acidic pH, anti-angiogenic factors, ionizing radiation, increased Ca^2+^ concentration, or heparanase activity are considered to stimulate the release of TEX. The protein cargo of TEX can be distinguished between luminal and membrane-bound molecules. The membrane-associated factors may include CD63, CD9, CD81, intercellular adhesion molecule (ICAM), epithelial adhesion molecule (EpCAM), CD44, FasL, programmed cell death protein 1 (PD-1), TNF-related apoptosis-inducing ligand (TRAIL), while TEX encapsulate proteins such as programmed cell death 6-interacting protein (ALIX), Rab proteins, dynamine, lysosome associated membrane proteins (LAMPs); signaling pathway components such as mitogen-activated protein kinase (MAPK), Rho, extracellular signal-regulated protein kinases 1 and 2 (ERK1/2), Wnt; cytoskeletal proteins such as actin and tubulin; and proangiogenic factors such as vascular endothelial growth factor (VEGF), basic fibroblast growth factor (bFGF), transforming growth factor-β (TGF-β).

As mentioned above, the cargo transported by TEX can be distinguished between intraluminal and surface-bound molecules. It was demonstrated, that TEX carry immunosuppressive ligands on their surface such as programmed cell death protein 1 (PD-1), Fas, and TNF-related apoptosis-inducing ligand (TRAIL) [28]. Moreover, the membrane of TEX is enriched in adherent molecules such as intercellular adhesion molecule (ICAM), epithelial cell adhesion molecule (EpCAM) and CD44. Furthermore, the membrane-associated cargo of TEX may contain transmembrane receptors such as chemokine receptor CXC type 4 (CXCR-4), c-MET, heat shock proteins, and above-mentioned tetraspanins, which are commonly used as sEV markers [1,29]. The lumen of TEX incorporates transport proteins such as programmed cell death 6-interacting protein (ALIX), Rab proteins, dynamin, and lysosome associated membrane proteins (LAMPs) and signaling molecules including mitogen-activated protein kinase (MAPK), Rho, extracellular signal-regulated protein kinases 1 and 2 (ERK1/2), Wnt, and even cytoskeletal proteins such as actin and tubulin, as indicated in Figure 2 [1]. The cargo components of sEVs and their biological effects in relation to their cellular origins are summarized in Table 1.

Different studies demonstrate that TEX are involved in inducing or promoting the process of new blood vessel formation during all stages of tumor development [12,27,48]. The analysis of TEX showed that cargo components also include factors that are essential for angiogenic pathways. Among these factors are pro-angiogenic growth factors such as basic fibroblast growth factor (bFGF), vascular endothelial growth factor (VEGF), and transforming growth factor-β (TGF-β), which were shown to have a strong impact on angiogenesis [6]. Moreover, TEX carry many other proteins that play a role in angiogenic processes including angiogenin, interleukin-6 (IL-6), IL-8, TIMP metallopeptidase inhibitor 1 (TIMP-1), and E-cadherin [27,49]. The clinical relevance of the pro-angiogenic cargo of TEX was supported in a recent paper showing that TEX carry a specific isoform of VEGF on their surface. Interestingly, this isoform preferentially localizes on the surface of TEX through its high affinity for heparin and has a profoundly-increased half-life compared to soluble VEGF. Additionally, this TEX-associated VEGF is not neutralized by bevacizumab and high levels were associated with disease progression in bevacizumab-treated cancer patients [33]. These results indicate that TEX may increase the resistance to anti-angiogenic therapies or may even interfere with these therapies.

The release of TEX is highly dependent on environmental conditions. Various signals have been described to influence the release of sEVs by tumor cells. One of the well-established triggers for the enhanced release of TEX, which is also a common characteristic of the TME, is hypoxia. It was shown that hypoxic conditions induce the release of TEX in breast cancer, bladder cancer, prostate cancer, head and neck cancer, and many other malignant entities [11,34,35,50]. An acidic environment is considered to be another important trigger for TEX release and is provided by increased anaerobic metabolism of tumor cells. Moreover, it was shown that higher levels of acidity and TEX release are strictly related to and strongly contribute to malignant tumor phenotypes [51]. Furthermore, TEX release may be induced by anti-angiogenic factors, ionizing radiation, increased Ca^2+^ concentration, or active forms of heparanase, as illustrated in Figure 2 [6,36,37,52,53,54]. It was recently shown that the cargo composition of TEX can be altered depending on the environmental factors which trigger the release of TEX. In particular, anti-cancer therapies were reported to promote the release of TEX, which are enriched in immunosuppressive or pro-angiogenic cargo components and ultimately weaken response to therapy or promote metastasis [55]. TEX generated under hypoxic conditions were shown to be especially enriched in pro-angiogenic factors, bearing a greater potential to contribute to new blood vessel formation [30]. Several pro-angiogenic cargo components of TEX were found to be regulated by hypoxia, including TGF-β, VEGF, lysyl oxidase homolog 2 (LOXL2), IL-8, insulin like growth factor binding protein 1 (IGFBP1), IGFBP3, and IGFBP5 [12].

## 4. Interactions of TEX with Recipient Cells

TEX are considered to be an important contributor to intercellular communication mechanisms and facilitate the tumor/stroma crosstalk. It was shown that this crosstalk allows the reprogramming of stroma cells and ultimately shapes a tumor-promoting microenvironment [28]. This microenvironment is characterized by the presence of pro-angiogenic factors, which are either secreted by the tumor cells or other cells present in the TME. TEX might carry a fraction of these pro-angiogenic factors and might also be an important regulator for the release of pro-angiogenic factors by other cells in the TME. Therefore, the interactions of TEX with recipient cells are of major importance to understanding the role of TEX in angiogenesis.

Several mechanisms have been described concerning the TEX/recipient cell interaction. Most data focusses on the internalization of TEX by recipient cells which can lead to the generation of a functional protein by delivery of nucleic acids [27,56]. The detailed mechanism for TEX uptake varies depending on the cargo of TEX and the recipient cell, as well as microenvironmental conditions [56]. The most-described pathways for the internalization of TEX are endocytosis, macropinocytosis, phagocytosis, and membrane fusion. Interestingly, most cell types are able to utilize several of these pathways to internalize TEX and blocking of one pathway might enhance the uptake of TEX by another pathway. Therefore, the uptake mechanisms need to be carefully studied and investigated for each cell type. For endothelial cells (ECs) it was shown, that TEX are internalized rapidly within 4 h of co-incubation and the most dominant internalization pathway was described to be endocytosis [35]. In other cell types, for example macrophages, phagocytosis is the most dominant uptake mechanism and macrophages are even more efficient in internalizing TEX compared to ECs [57]. However, it was reported that macrophages internalize TEX not only via phagocytosis, but also via clathrin- and caveolin-dependent endocytosis and macropinocytosis [56]. T cells were shown to internalize only minimal quantities of TEX, indicating that other forms of interaction are responsible for the TEX-mediated effects on this cell type [58].

Surface-mediated receptor–ligand interactions are another form of interaction between TEX and recipient cells. TEX carry ligands on their surface, which can bind to receptors expressed by the recipient cells and initiate a signaling cascade. This was shown for multiple cell types, including ECs. Reported signaling pathways are, among others, the notch pathway [59], the adenosine pathway [25], ephrin pathway [38], and the E-cadherin pathway [49].

TEX also carry functionally-active enzymes on their surface which can produce factors that stimulate surrounding cells in a paracrine fashion. This was shown for the ectonucleotidases CD39 and CD73, which generate adenosine and stimulate EC growth depending on adenosine A_2B_ receptor signaling [25].

## 5. Effects of TEX on Endothelial Cells

Most reports which focus on the pro-angiogenic effects of TEX describe the interactions of TEX and ECs. Interestingly, it was shown for multiple types of cancer, that TEX induce a pro-angiogenic phenotype in ECs and stimulate their proliferation, migration, and tube formation [12]. These functional alterations of ECs were described to be mediated via different pathways. It was demonstrated that TEX carry ephrin type B receptor 2 (EPHB2) and that EPHB2 promotes angiogenesis by ephrin-B reverse signaling, inducing STAT3 phosphorylation. Accordingly, a STAT3 inhibitor was presented as a strategy to inhibit TEX-induced angiogenesis [38]. Another study demonstrated that the underlying mechanism for the pro-angiogenic effects of TEX is adenosine A2B receptor signaling [25]. The transmembrane glycoprotein podoplanin was also suggested to play a crucial role in mediating effects of TEX on ECs [39]. Besides that, the delivery of nucleic acids to ECs by TEX is considered to promote pro-angiogenic functions of ECs [27]. In particular, miRNAs in the lumen of TEX were described to be involved in reprogramming ECs. Most frequently, the following miRNAs appeared in the literature and seem to play an important role in angiogenic processes—miR-21, miR-23a, miR-30b, miR-126a, and miR-210 [12]. However, other RNA classes were also described in the same context. TEX derived from colorectal cancer cells are enriched with mRNAs such as CDK8, ERH, and RAD21, which are mainly related to the cell cycle [40]. Also, TEX-associated long noncoding RNAs (lncRNAs) were reported to promote angiogenesis. The transfer of long intergenic noncoding RNA CCAT2 to ECs by TEX enhanced in vitro and in vivo angiogenesis and upregulated VEGFA and TGF-β levels on ECs [41]. It seems that the pro-angiogenic effects of TEX on ECs are orchestrated via multiple pathways which probably converge, therefore, making TEX efficacious promotors of angiogenesis.

Besides these direct effects of TEX on ECs, it was shown that reprogrammed ECs secrete several potent growth factors and cytokines and stimulate pericyte PI3K/AKT signaling activation and migration [30]. Additionally, it was demonstrated that ECs also release sEVs themselves which can impact the process of new blood vessel formation [60]. These stroma-cell-derived small extracellular vesicles (EC-derived sEVs) in human plasma carried vascular cell adhesion molecule-1 (VCAM-1), endothelial nitric oxide synthase, von Willebrand factor (vWF), platelet derived growth factor BB (PDGF-BB), large neutral amino acid transporter (LAT-1), angiopoietin 1 and 2, glucose transporter 1 (GLUT-1), and lysyl oxidase homolog 2 (LOXL-2) [61]. The cargo of EC-derived sEVs also consists of nucleic acids, such as miR-214, which can be transferred to other ECs and stimulate their migration and angiogenesis. sEVs from miR-214-depleted ECs fail to stimulate these processes [42]. However, only limited data are available regarding EC-derived sEVs and their role in tumor angiogenesis or tumor progression. Future studies are necessary to analyze the detailed cargo composition of sEVs derived from tumor-associated ECs as well as studying their functions in the TME.

## 6. Effects of TEX on Macrophages

Tumor-associated macrophages (TAMs) constitute a plastic and heterogeneous cell population of the TME that can account for up to 50% of some solid neoplasms. Most often, TAMs support disease progression and resistance to therapy, however, TAMs can also mediate antineoplastic effects, especially in response to pharmacological agents that boost their phagocytic and oxidative functions [62]. The phenotype of TAMs is heterogenous and translates into distinct functions in the TME. TAMs can be categorized into two subsets, classically-activated (M1) and alternatively-activated (M2) macrophages based on their phenotype and distinct functional abilities. In the TME, the M2 phenotype is considered to be the dominant macrophage population and is responsible for tumor progression and associated with poor prognosis [43,63]. The polarization towards M1 or M2 relies on the presence of different stimuli. Macrophages exposed to cytokines like IL-12, TNF, or IFNγ, microbe-associated molecular patterns (MAMPs) such as bacterial lipopolysaccharide (LPS), or other toll-like receptor (TLR) agonists acquire an M1 state. Conversely, IL-4, IL-5, IL-10, IL-13, CSF1, TFG-β, and PGE2 promote macrophage polarization towards an M2 state [62]. Some of these stimuli, such as TGF-β, were shown to be part of the TEX cargo [44,45], therefore, it was suggested, that TEX are involved in polarizing macrophages. Glioblastoma-derived TEX were shown to polarize macrophages towards the M2 phenotype, whereas melanoma and head- and neck-cancer-derived TEX were shown to induce a mixed M1/M2 phenotype of macrophages [25,43,57]. These results indicate that TEX are indeed involved in macrophage polarization, however, the direction of this polarization is dependent on the composition of TEX. It was also demonstrated that TEX are able to stimulate the recruitment of macrophages [64] and injection of TEX-containing plugs into mice promoted a massive infiltration of M2 macrophages [25]. The reprogramming of macrophages by TEX was also connected to the formation of a pre-metastatic niche, which is considered to be one of the hallmarks of TEX functions [64].

**Figure 3:**
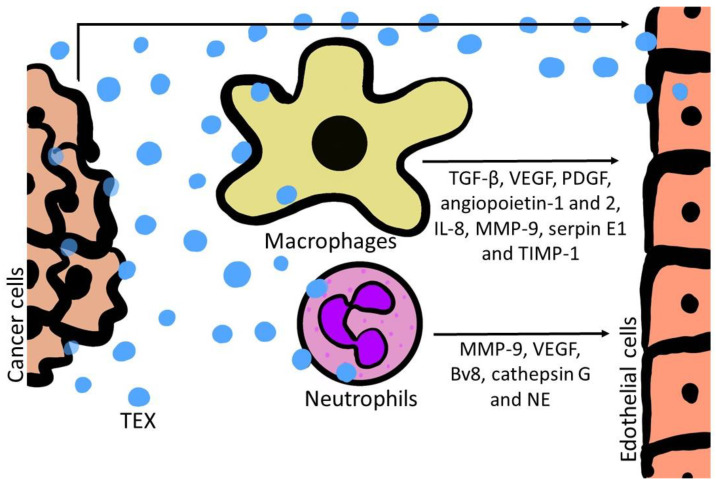
A schematic visualizing the reprogramming of endothelial cells by TEX. TEX carry a variety of pro-angiogenic factors, which are either surface-bound or encapsulated in their lumen. TEX interact directly with endothelial cells or reprogram other cells in the tumor microenvironment such as macrophages and neutrophils and promote the release of pro-angiogenic factors to stimulate a pro-angiogenic phenotype in endothelial cells.

One important effect of TAMs is the provision of trophic and nutritional support for cancer cells or other cells in the TME, ultimately promoting disease progression. This also includes the stimulation of angiogenesis and, therefore, the crosstalk between TAMs and ECs. M2-like TAMs were described as key effectors of stimulating angiogenesis, since they produce diverse pro-angiogenic factors, like TGF-β, VEGF, PDGF, and angiogenic chemokines, as indicated in Figure 3 [65]. Macrophage infiltration in tumors is generally associated with high vascular density and M2-like TAMs predominantly localize in hypoxic tumor areas [66]. As indicated above, TEX are able to reprogram macrophages and, therefore, might promote the pro-angiogenic effects of TAMs. Recent work supports this concept and shows that TEX not only directly interact with ECs to stimulate new blood vessel formation, but also interact in an indirect way by reprogramming macrophages towards a pro-angiogenic phenotype [67]. In head and neck cancer it was demonstrated that TEX stimulate the release of pro-angiogenic factors by macrophages and therefore stimulate ECs in an indirect way. Especially angiopoietin-1 and 2, IL-8, MMP-9, serpin E1, and TIMP-1 were reported to be released by macrophages in larger quantities after co-incubation with TEX [25]. This reprogramming of macrophages towards a pro-angiogenic M2 phenotype by TEX can be either induced by proteins such as TGF-β and nucleic acids such as miRNAs, or other TEX-associated factors such as adenosine [25,68].

It is also important to mention that reprogrammed TAMs again release sEVs with tumor-promoting functions. Recent studies reported a functional role for miRNA-containing sEVs derived from M2-like macrophages in regulating migration and invasion of colorectal cancer cells [69]. Another study demonstrated the sEV-mediated transfer of functional apolipoprotein E from TAMs to tumor cells, resulting in an enhanced migration of gastric cancer cells [46]. Arginase-1(+) sEVs derived from TAMs promoted tumor cell migration and proliferation in glioblastoma and were considered as a distributor of proteins with pro-tumor functions in the TME [43]. Although no evidence exists so far, it is likely that TAM-derived sEVs also carry a pro-angiogenic cargo, which reflects the cytosolic contents and cell-surface molecules associated with the M2-like phenotype. Analog to TEX, the sEVs derived from TAMs might also be involved in stimulating ECs and inducing or promoting angiogenesis. However, future studies are necessary to confirm this hypothesis.

## 7. Effects of TEX on Neutrophils

As described above, the M2-like phenotype of macrophages contributes to tumor progression by induction and stimulation of angiogenesis through the secretion of pro-angiogenic factors [70]. Analog to macrophages, it was reported that neutrophils can play a similar role, however, much less is currently known about their pro-tumor effects. It is commonly observed, that patients with solid tumors show elevated levels of neutrophils compared to healthy individuals [71]. Neutrophils are the most abundant type of granulocytes and form part of the polymorphonuclear cell family (PMNs). They are an essential part of the innate immune system, with being one of the first responders of inflammatory cells to migrate towards the site of inflammation or tissue damage, acting against a wide variety of pathogens [72]. Defining the exact roles of neutrophils is still ongoing research, however, they are considered to participate in the immune response through the regulation and recruitment of other immune cells, such as monocytes/macrophages and dendritic cells [73], as well as through modulation of the interaction between B and T cells [72]. Analogous to macrophages, neutrophils show heterogenous phenotypes which allow them to be proficient in distinct functions [74]. Compared to M1 and M2 macrophages, neutrophils have an activation spectrum that has not yet been clarified, however, they can be grouped according to the same logic—N1 (pro-inflammatory polarization) and N2 (anti-inflammatory polarization) [72]. The N2 phenotype is acquired by the presence of TGF-β, favoring the infiltration of neutrophils with high expression of CXCR-4, vascular endothelial growth factor A (VEGF-A), and metalloprotease 9 (MMP-9) [75]. Neutrophils are efficacious producers of pro-angiogenic factors and especially cells with the N2 phenotype contribute to angiogenesis and tissue invasion, ultimately promoting tumor progression [76,77,78]. Although it is still ongoing research to define the role of TEX in neutrophil polarization, TEX carry factors that are considered important for a polarization towards the N2-like phenotype. These factors include TGF-β, MMP-9, and arginase-1 [35,43,44]. Evidence for functional reprogramming of neutrophils by TEX was reported in a recent paper showing that gastric-cancer-cell-derived sEVs enhanced PD-L1 expression of neutrophils, thereby suppressing the activity of T cells [47]. The concept that TEX interact with neutrophils and promote their activation is also supported by co-incubation studies leading to both pro-inflammatory [79] and anti-inflammatory profiles [80,81] depending on the source and cargo components of TEX. One effector for the promotion of pro- or anti-inflammatory responses by neutrophils is the release of granules [72]. It should be noted that the neutrophil secondary granules are enriched in MMP-9, which is a decisive component of angiogenic pathways [75,78,82]. Interestingly, neutrophils are the only cell type that is capable of releasing MMP-9 without being attached to its physiological inhibitor TIMP-1. Therefore, the secretion of highly-active MMP-9 by neutrophils can have a major impact on angiogenic sites [83]. Additionally to MMP-9, neutrophils release other potent growth factors to promote angiogenesis, such as VEGF or Bv8, as indicated in Figure 3 [75,77,82,84,85]. It was observed that neutrophils generate heterogenous microparticles, which are capable of altering epithelial gene expression as well as cell proliferation in HUVECs [86].

Another activity of neutrophils in the TME is the formation of extracellular traps (NETs), which can be stimulated by tumor cells [87]. NETs consists of nuclear contents, combined with cytosolic proteins and granules [83]. Among these components, cathepsin G, elastase (NE), and MMP-9 are most frequently described in the literature due to their functional relevance. It was demonstrated that the release of NE and cathepsin G activates platelets, which in turn activate the coagulation cascade through inactivation of the anticoagulant protein factor pathway inhibitor [88,89]. Thus, the formation of NETs is essential for accelerating the process of thrombus formation in solid tumors. A study with sEVs derived from breast cancer cells highlighted that TEX are able to induce the release of NETs and accelerate thrombi formation. These are significant findings since the crosstalk between TEX and neutrophils might play a major role in the establishment of cancer-associated thrombosis [90].

To conclude, it is well established that neutrophils are involved in promoting angiogenesis and interacting with ECs in the TME. It has also been shown that TEX play a substantial role in activating neutrophils and promoting their functional activity. However, the link between angiogenesis and neutrophils, which were reprogrammed by TEX, is still ongoing research. Future studies are necessary to focus on the detailed mechanisms of the interaction between neutrophils and TEX, with special regards to their pro-angiogenic functions.

## 8. Conclusions

Research in recent years had a strong focus on TEX and uncovered multiple effects of sEVs in the TME. One hallmark of TEX is the induction and promotion of angiogenesis, which is probably orchestrated by several signaling pathways, depending on the cargo composition of TEX and the recipient cells, as well as environmental factors. Interestingly, TEX do not only directly communicate with ECs and reprogram them to an angiogenic phenotype, they also interact with other cells in the TME that, in response, contribute to tumor angiogenesis. Defining these direct and indirect pathways to determine whether pharmacologic treatments, chemo- or radiotherapy, or exposure to compounds may influence the amount and functionality of TEX is an area of substantial interest. Blocking TEX-mediated effects may be a promising strategy to overcome therapy resistance to anti-angiogenic therapies or reduce tumor vascularization to ultimately ameliorate disease progression.

## Figures and Tables

**Table 1:** Comparison of articles from our literature search with emphasis on source of TEX or sEVs, their cargo, and biological effects. Abbreviations: IL: Interleukin; TRAIL: TNF-related apoptosis-inducing ligand; PD-L1: Programmed death-ligand 1; HSC70: Heat shock 70 kDa protein 8; PDGF: Platelet-derived growth factor; VEGF: Vascular endothelial growth factor; Vash1: Vasohibin-1; Angpt1: Angiopoietin 1; Flk1: Fetal liver kinase 1; uPA: Urokinase-type plasminogen activator, MMP-9: Matrix metallopeptidase 9; IGFBP3: insulin like growth factor binding protein 3; HGF: Hepatocyte growth factor; EPHB2: ephrin type B receptor 2; TGF-β: Transforming growth factor-β; STAT3: Signal transducer and activator of transcription 3.

Reference	Cancer Type/Source of sEVs	TEX Cargo	Tumor-Promoting Biological Effects
Skog et al. [27]	Glioblastoma	Angiogenin, IL-6, IL-8	Angiogenesis
Sharma et al. [7]	Melanoma	FasL, TRAIL, PD-L1	Immunosuppression
Ludwig et al. [25]	Head and neck squamous-cell carcinoma cell line UMSCC47	CD39/CD73, adenosine	M2 macrophage Polarization and enhanced secretion of angiogenic factors
Umezu et al. [26]	Leukemia cells (K562)	miR-92a	Enhanced endothelial cell migration and tube formation
Kucharzewska et al. [30]	Glioma cells	Matrix metalloproteinases, IL-8, PDGFs, caveolin 1, and lysyl oxidase	Activation of vascular cells
Ko et al. [33]	ES2, HCT116, and 786-0 cell lines	Heparin-bound VEGF on the surface of sEVs	Endothelial cells migration and tube formation
Xue et al. [34]	adipose Mesenchymal stem cells	Vash1, Angpt1 and Flk1	enhancement of Angiogenesis through the PKA-signaling pathway
Ludwig et al. [35]	Head and neck squamous-cell carcinoma cell lines (PCI-13, UMSCC47)	uPA, MMP-9, coagulation factor III, thrombospondin-1, uPA, IGFBP-3, endostatin	Reprogramming of HUVECs
Thompson et al. [36]	Bacterial heparinase-III-treated CAG, ARH-77, MDA-MB-231	Syndecan-1, VEGF, and HGF	Enhanced endothelial cell invasion
Zeng et al. [37]	Hepatocellular carcinoma	VEGF	Tumor vasculogenesis despite anti-angiogenic therapy
Sato et al. [38]	Head and neck squamous cell carcinoma	EPHB2	Promotion of angiogenesis
Carrasco-Ramirez et al. [39]	Melanoma	Podoplanin	Modulation of lymphatic vessel formation
Hong et al. [40]	Colorectal cancer	Cell-cycle--related mRNAs	Proliferation of endothelial cells
Lang et al. [41]	Glioma cells	Long non-coding RNA CCAT2	Promotion of angiogenesis and inhibition of endothelial cell apoptosis
Van Balkom et al. [42]	Human microvascular endothelial cell line (HMEC-1)	miR-214	Prevention from cell cycle arrest and, thus, stimulation of blood vessel formation
Azambuja et al. [43]	Reprogrammed macrophages	Arginase-1	Glioblastoma progression
Webber et al. [44]	Prostate, bladder, colorectal and breast cancer cell lines	TGF-β	Differentiation of fibroblasts to myofibroblasts
Hong et al. [45]	Acute myeloid leukemia	TGF-β	Immunosuppression
Zheng et al. [46]	Tumor-associated macrophages	Apolipoprotein-E	Migration of gastric cancer cells
Shi et al. [47]	Gastric cancer cells	STAT3	PD-L1 expression on neutrophils to suppress T-cell-mediated immunity
